# Investigation of the Adaptivity Degree of State Variables in Linear Models of Dynamic Systems

**DOI:** 10.3390/s26020354

**Published:** 2026-01-06

**Authors:** Maria Selezneva, Konstantin Neusypin, Anastasia Surkova

**Affiliations:** Department of Information and Control Systems (IU1), Bauman Moscow State Technical University, Moscow 105005, Russia; neysipin@mail.ru (K.N.); a.d.surkova@mail.ru (A.S.)

**Keywords:** adaptability degree, referred noise, aircraft, inertial navigation system, qualitative analysis

## Abstract

This study investigates the concept of the adaptability degree of state variables in mathematical models used in estimation algorithms. Two numerical criteria are proposed for calculating the adaptability degree of state variables in linear models. Qualitative characteristics of the adaptability of inertial navigation system errors are studied under conditions of varying sampling periods and flight altitudes of an atmospheric aircraft. The developed methods allow for the creation of models for estimation algorithms with desired adaptability properties.

## 1. Introduction

Various information-processing methods often involve mathematical models of dynamic objects and systems. These models possess diverse qualitative characteristics that influence their effectiveness in algorithms and software systems. Endowing models with desired properties enhances their quality for specific practical applications. Algorithms using models that have improved quality characteristics are successfully applied in modern technology [1,2,3].

Control of dynamic objects requires preliminary investigation into the feasibility of implementing the control process. Various criteria are used to determine the controllability of an object, with the most common being the Kalman controllability criterion [4,5]. In many tasks, such as aircraft and spacecraft applications, automotive control, and chemical process control, it is often necessary not only to ensure controllability but also to obtain information about the qualitative characteristics of the controlled object [1,2]. Control relies on information about the object’s state and frequently employs its mathematical model [6,7].

Observability characterizes the ability to determine an object’s state based on available measurements [8]. In practice, the Kalman observability criterion is commonly used to determine the observability of systems. Observation quality affects control efficiency and the accuracy of measurement systems being corrected, such as aircraft navigation complexes (NCs) [9,10].

Mathematical models used for control and estimation are derived from physical, chemical, and other functional laws of the studied object. When objects are in use [11], model parameters are subject to changes; therefore, various parametric identification algorithms are used to increase the adequacy of models [12]. For example, re-entering aircraft in control and navigation systems use models that include the parameter *g*—gravitational acceleration. The parameter *g* increases as the aircraft descends and is subject to identification [13].

The models describing the object under study are characterized by the properties of controllability, observability, and identifiability, which are structural properties of systems. Well-known criteria are used to determine these characteristics [14].

In practical applications, it is often necessary to conduct qualitative assessments of these properties: How effective is the control of the object? How well can the object be observed based on the selected measurements? How quickly and with what accuracy can parametric identification of the object model be performed? To answer these questions, various qualitative characteristics of the models are used.

Researchers have solved the questions of how to select the interval for the most effective aircraft control, determine the number and arrangement of actuators in spacecraft control systems, establish the required measurement duration to obtain consistent estimates of the directly unmeasured state variables for inertial navigation system (INS) error correction, and identify achievable accuracy levels for parameter identification in models of dynamic, mechanical, and biological systems, manufacturing and chemical processes, and models in ecology, medicine, and social and political systems. Various diagrams and quantitative and qualitative criteria have been reported as a result [1,2,3]. When studying dynamic objects and processes, other qualitative model characteristics are also employed to gain comprehensive understanding, such as sensitivity and stability margins, which have been thoroughly developed and well-documented in [15,16,17]. However, there are also other, less studied model characteristics, including dynamicity degree [18,19] and adaptability degree [20,21].

Existing criteria for adaptability metrics have been developed for specific problems. The adaptability degree criterion for multi-agent resource management systems [21] is based on calculating the difference between the average and minimum satisfaction of system agents after solution stabilization and the minimum satisfaction value immediately after the impact (before processing the changes). The criterion also includes the simulation time step and the rebalancing time. This criterion is only applicable to multi-agent systems and determines the speed of the transition process, which allows the system state to be restored in the event of a disturbance.

The concepts of the observability and adaptability degrees are very close. When assessing the observability degree, the primary focus is on the estimation accuracy achieved within a limited observation time. The adaptability degree characterizes the speed of the system’s response to changes, assuming that the specified estimation accuracy is maintained. Therefore, in semantic terms, the adaptability degree better reflects the proposed criterion.

Unlike the adaptability degree, the parametric identifiability degree characterizes the efficiency of determining the parameters of the model matrix and is not related to state variables. The identifiability degree of state variables is rarely used and is identical to the concept of the observability degree.

At the current stage of designing and operating complex systems of highly dynamic objects, the use of algorithms and mathematical models with high adaptability degrees becomes relevant. For example, during high-intensity maneuvering in aircraft, it is recommended to use adaptive models that promptly account for system parameter changes and control algorithms providing adaptation to changing flight regimes, compensation of external disturbances, and precise execution of control commands. During carrier-based aircraft landing operations, strong airflow disturbances may occur due to the island superstructure on the flight deck.

To prevent emergency situations, the aircraft must have a rapid response to airflow disturbances. In the control system of highly maneuverable aircraft and the measurement complex correction system, adaptive algorithms and models with enhanced adaptability properties are employed. By analogy with controllability, observability, stability, and similar concepts, adaptability represents a system’s fundamental ability to respond to events. The degree of adaptability is a qualitative characteristic of the adaptation process that determines how effectively the adaptation can be implemented. To increase the adaptability degree of algorithms, rigid and/or additional measurement linkages are employed [22,23]. The more information about the situational changes or event occurrences is used in an algorithm, the higher its adaptability degree.

This study investigates the adaptability degree of not algorithms, but state variables in models employed by such algorithms. Developing models with enhanced adaptability degrees remains challenging due to the absence of established criteria for determining this characteristic. Developing adaptability degree criteria for models and their state variables is an urgent task. The choice of models with improved adaptability properties is based on practical considerations and requires a large number of expensive experiments.

Section 1 presents an adaptive Kalman filter and a measurement transformation method used to develop numerical criteria for adaptability degree assessment.

Section 2 introduces two original numerical criteria for evaluating the adaptability degree of state variables in linear dynamic system models.

Section 3 investigates the adaptability degrees of INS errors, with the performance characteristics of INS I-11 used as a case study.

Section 4 presents the discussion of the study’s results.

In summary, this paper proposes two criteria for the adaptability degree of state variables in linear dynamic system models. The application of these criteria is demonstrated through an example of determining the adaptability degree of an INS error model.

## 2. Adaptive Estimation Algorithm and Formation of Referred Measurements for Linear Models

One of the little investigated qualitative characteristics of models and state vector components is the adaptability degree of a model, which is an abstract characteristic. Existing approaches presented in [20,21] allow us to empirically determine which of the models used in the algorithm adapt faster to changes in the studied process. These approaches provide only a relative estimation of a system model’s adaptability and do not enable comparisons of adaptability degrees among state vector components across different models. Therefore, all known approaches are inconvenient when comparing the quality of adaptation in general.

We investigate the adaptability property of models used in the estimation algorithms. Estimation algorithms are widely employed in aircraft control systems and measurement complexes. Therefore, using models with enhanced adaptability characteristics improves the effectiveness of control systems and measurement complexes. This improvement is achieved through more accurate estimates of state variables for systems and complexes. Adapting to changes allows for faster reaction and takes into account external and internal changing factors.

Let the object be described by the following equation:

(1) $$x_{k}=\Phi x_{k-1}+Gw_{k-1}$$

where $x_{k-1}$—state vector; $w_{k-1}$—input noise vector, representing a discrete analog of zero-mean white Gaussian noise; Φ(n×n)—system matrix; and G(n×l)—input noise matrix.

A part of the state vector is measured:

(2) $$z_{k}=Hx_{k}+v_{k}$$

where $z_{k}$—*m*-dimensional measurement vector; H(m×n)—measurement matrix; and $v_{k}$—m-dimensional measurement noise vector, representing a discrete analog of zero-mean white Gaussian noise, with v and w being mutually uncorrelated (i.e., $M\;\left[v_{j}w_{k}^{T}\right]=0$).

The Kalman filter equations are as follows:

(3) $$\begin{array}{cc} \; & {\hat{x}}_{k+1}=\Phi {\hat{x}}_{k}+K_{k+1}v_{k+1};\\ \; & P_{(k+1)/k}=\Phi P_{k}\Phi^{T}+Q_{k};\\ \; & K_{k+1}=P_{(k+1)/k}H^{T}{\left[HP_{(k+1)/k}H^{T}+R_{k+1}\right]}^{-1};\\ \; & P_{k+1}=\left(I-K_{k+1}H\right)P_{(k+1)/k}, \end{array}$$

where $P_{(k+1)/k}$ is the a priori estimation error covariance matrix; $P_{k+1}$ is the a posteriori estimation error covariance matrix; $K_{k+1}\in R^{n\times m}$ is the filter gain matrix; and $v_{k+1}=z_{k+1}-H\Phi {\hat{x}}_{k}$ is the innovation sequence.

The Kalman filter not only reconstructs the entire system state vector but also suppresses the measurement noise influence:

(4) $$\begin{array}{cc} \; & M\left(w_{k}\right)=0;M\left(w_{k}w_{j}^{T}\right)=Q_{k}\delta_{kj};\\ \; & M\left(v_{k}\right)=0;M\left(v_{k}v_{j}^{T}\right)=R_{k}\delta_{kj}. \end{array}$$

It is assumed that the initial system state, disturbance vector, and measurement error are mutually uncorrelated:

(5) $$M\left(x_{0}w_{k}^{T}\right)=0;M\left(x_{0}v_{k}^{T}\right)=0;M\left(w_{k}v_{k}^{T}\right)=0.$$

The optimal state vector estimate is determined by the first equation from (3).

Adaptive algorithms based on direct feedback from the innovation sequence provide an additional adaptation loop. This configuration enhances the algorithm’s adaptive properties. The filter gain matrix determines the weighting factor applied to the innovation sequence in the state vector estimate. Under ideal measurement conditions (i.e., absence of measurement noise), the gain matrix is set to maximum values. As measurement noise increases, the innovation sequence receives reduced weighting in the state vector estimation. An increase in the weight with which measurements are involved in forming an estimate also increases the adaptive properties of the algorithm but reduces the accuracy of the estimate and limits the reduction in *P*.

The adaptability degree of an estimation algorithm can be judged by the rate of estimation error reduction. The difference $P_{k}-P_{k-1}$ characterizes the algorithm’s rapid response but depends on $K_{k}$, and when *V* varies, it fails to provide an accurate assessment of the algorithm’s adaptability degree.

Let us consider the adaptability degree of the state variables of the model, for example, variables used in the Kalman filter.

Directly measured components of the state vector have the maximum adaptability degree since they contain information about changes in the process under study. The measurement quality and reliability of the directly measured state vector component depend solely on the measurement error (i.e., measurement noise). The magnitude of this noise is determined by passive and active interference sources. Consequently, when assuming maximal adaptability degree for the measured state vector component, we imply that it equals 100%—limited exclusively by measurement noise.

Without loss of generality, assume a single state vector component is measured, i.e., $H=\left[1\;0\;\cdots \;0\right]$.

We divide each measurement step into n substeps and express these measurements through the state vector at the first measurement substep:

(6) $$\begin{array}{cc} \; & z_{1}=Hx_{1}+v_{1};\\ \; & z_{2}=H\Phi x_{1}+Hw_{1}+v_{2};\\ \; & \vdots \\ \; & z_{n}=H\Phi^{n-1}x_{1}+H\Phi^{n-2}w_{1}+\cdots +Hw_{n-1}+v_{n}, \end{array}$$

or in matrix form

(7) $$z^{*}=Sx_{1}+v^{*},$$

where $z^{*}=\left[\begin{array}{c} z_{1}\\ z_{2}\\ \vdots \\ z_{n} \end{array}\right],S=\left[\begin{array}{c} H\\ H\Phi \\ \vdots \\ H\Phi^{n-1} \end{array}\right],v^{*}=\left[\begin{array}{c} v_{1}\\ Hw_{1}+v_{2}\\ \vdots \\ H\Phi^{n-2}w_{1}+\cdots +Hw_{n-1}+v_{n} \end{array}\right]$.

Let us express, from the system equation, the state vector at the first measurement substep:

(8) $$\begin{array}{cc} \; & x_{1}=S^{-1}z^{*}+S^{-1}v^{*}. \end{array}$$

Let us introduce the following notation:

(9) $$y=S^{-1}z^{*},$$

and write Equation (9) in scalar form:

(10) $$y^{i}=a_{1}z_{1}+a_{2}z_{2}+\cdots +a_{n}z_{n},$$

where $y^{i}$ denotes the *i*-th element of vector *y*; $a_{1},a_{2},\cdots ,a_{n}$ are the elements of the *i*-th row of matrix $S^{-1}$. $S^{-1}$ exists because *S* is the observability matrix.

For the remaining state vector components, the measurement equations are formulated according to Equation (10).

We introduce the concept of referred measurement noise [24]. For an arbitrary state vector component, the referred measurement noise is defined according to Equation (10) as

(11) $$v^{i*}=a_{1}v_{1}+a_{2}v_{2}+\cdots +a_{n}v_{n}$$

The variance in the measurement noise referring to the *i*-th component is determined by coefficients $a_{1},a_{2},\cdots ,a_{n}$:

(12) $$r^{*i}=M\left[v^{*i}\right]=\left[a_{1}^{2}+a_{2}^{2}+\cdots +a_{n}^{2}\right]r$$

where $r=M\;\left[v^{2}\right]$ is the variance of the original measurement noise v.

## 3. Criteria for Adaptability Degree of Model State Variables

Given that a measure represents a category expressing the dialectical unity of qualitative and quantitative object characteristics, the adaptability measure can be evaluated through two key characteristics: estimation accuracy and convergence time. The relationship between these characteristics is defined by the established formula [5,10]:

(13) $$P_{n}^{i}=\frac{P_{0}^{i}r^{i}}{NP_{0}^{i}+r^{i}},$$

where $P_{0}^{i}=M\;\left[{\left(x_{0}^{i}\right)}^{2}\right]$—the initial estimation error variance; $P_{n}^{i}$—the estimation error variance; $r^{i}$—the measurement noise variance of the directly measured state variable; and $N^{i}$—the computation step index.

Let us set the desired estimation accuracy for the *i*-th state vector component as 10% of the initial value *P*. Let us determine the number of computation steps required to estimate the component with this specified accuracy.

(14) $$N^{i}=\frac{9r^{i}}{P_{0}^{i}}$$

Similarly, we determine the number of steps required to estimate the directly measured state variable. Let us consider the ratio between the computation steps needed to estimate the investigated *i*-th component and those for the directly measured component.

The adaptability degree criterion is defined as

(15) $$\xi^{i}=\frac{P_{0}r^{i}}{P_{0}^{i}r}$$

where $P_{0}$ is the initial error variance of the directly measured state vector component.

When the initial estimation error variance cannot be precisely determined, the following adaptability degree criterion may be used:

(16) $$\eta^{i}=\frac{M\left[{\left(y^{i}\right)}^{2}\right]r^{*i}}{M\left[{\left(x^{i}\right)}^{2}\right]r}$$

where $M\;\left[{\left(y^{i}\right)}^{2}\right]$ is the variance of the directly measured state variable; $M\;\left[{\left(x^{i}\right)}^{2}\right]$ is the variance of an arbitrary *i*-th state vector component.

In adaptability degree criteria (15) and (16), observability is quantified by a scalar metric. This feature makes it possible to compare the degrees of adaptability of the components of different model state vectors.

The adaptability degree of model state variables may alternatively be determined through another method. We calculate the number of steps required for estimation accuracy to reach 10% of the initial estimation error value.

(17) $$P_{n}=\frac{P_{0}}{10}$$

The number of computation steps to achieve a specified estimation error level is determined as

(18) $$N_{n}^{i}=\frac{10-r^{*i}}{P_{0}}$$

This method defines the adaptability degree of state variables, which primarily depends on model properties and how strongly the linkage with measurements is implemented in the model.

## 4. Study of Adaptability Degree of INS Error Model State Variables

Let us consider the adaptability degree of state variables in the INS error model, which serves as the primary measurement system for the NC. The application of linear filtering methods for INS correction represents the most common approach to improve navigation data accuracy.

When the INS operates in correction mode from external measurement systems, compensation of its errors is usually carried out using estimation algorithms. In the INS correction mode, errors are estimated and then compensated in the output signal of the system [24,25].

**Figure 1:**
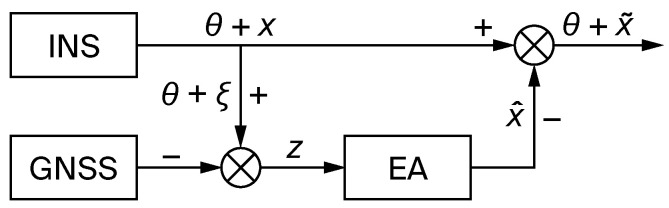
INS correction scheme with estimation algorithm: GNSS—global navigation satellite system; EA—estimation algorithm; θ—true navigation information; *x*—INS error vector; ξ—GNSS error vector; *z*—measurement vector; $\hat{x}$—estimation of the INS error vector; $\tilde{x}$—INS error estimations.

The INS correction scheme from an external measurement system with an estimation algorithm is shown in Figure 1.

In addition to the difference in the time intervals required for a satisfactory estimation of the INS errors, the relative estimation errors in relation to the estimated nominal value are also different. In this regard, the question about the adaptability degree of various INS errors is posed.

The adaptability degree of a state vector component is a qualitative characteristic of its determination from the measured signal over a finite time interval.

The INS error equations are as follows [10,23,24]:

(19) $$\begin{array}{cc} \; & \delta V_{k}=\delta V_{k-1}-gT\varphi_{k-1};\\ \; & \varphi_{k}=\varphi_{k-1}-\frac{T}{R}\delta V_{k-1}+T\varepsilon_{k-1};\\ \; & \varepsilon_{k}=T\varepsilon_{k-1}, \end{array}$$

where *T*—the sampling period, *R*—the radius of the earth, *g*—gravity acceleration, $\delta V_{k}$—INS velocity error; $\varphi_{k}$—the deviation angle of the gyrostabilized platform (GSP) relative to the moving trihedron; and $\varepsilon_{k}$—the GSP drift rate. In this study, the measurement equation has the form from (2), $H=\left[\begin{array}{ccc} 1 & 0 & 0 \end{array}\right]$.

We form the measurement vector as follows:

(20) $$y=S^{-1}\left[\begin{array}{c} \delta V_{k}\\ \delta V_{k+1}\\ \delta V_{k+2} \end{array}\right]$$

where $S=\left[\begin{array}{ccc} 1 & 0 & 0\\ 1 & -gT & 0\\ 1+\frac{gT^{2}}{R} & -2gT & -gT^{2} \end{array}\right],\Phi =\left[\begin{array}{ccc} 1 & -gT & 0\\ \frac{-T}{R} & 1 & T\\ 0 & 0 & 1 \end{array}\right]$.

Then, for the direct measurement of the state vector components, we obtain the following equations:

(21) $$\begin{array}{cc} \; & z\left(\delta V\right)=z_{k};\\ \; & z\left(\varphi \right)=\frac{1}{gT}z_{k}-\frac{1}{gT}z_{k+1};\\ \; & z\left(\varepsilon \right)=-\frac{1}{gT^{2}}z_{k}-\frac{1}{R}z_{k}+\frac{2}{gT^{2}}z_{k+1}-\frac{1}{gT^{2}}z_{k+2}. \end{array}$$

These equations are obtained in accordance with (20). We define the variance of the measurement noise referred to the angle of deviation of the gyrostabilized platform relative to the moving trihedron:

(22) $$r_{2}^{*}=\frac{2}{g^{2}T^{2}}r,$$

where *r*—the variance of the velocity measurement error, which is measured directly by the external information sensor. Based on criterion (16), the determination of the adaptability degree for the second component will be carried out in accordance with

(23) $$\eta_{2}=\frac{M\left[\delta V^{2}\right]2}{M\left[\varphi^{2}\right]g^{2}T^{2}}$$

The adaptability degree of the GSP drift rate is defined similarly:

(24) $$\eta_{3}=\frac{M\left[\delta V^{2}\right]\left[\frac{5}{g^{2}T^{4}}+{\left(\frac{1}{gT^{2}}+\frac{1}{R}\right)}^{2}\right]}{M\left[\varepsilon^{2}\right]}$$

We substitute the numerical values of the parameters obtained as a result of a semi-natural experiment with a real I-11 system [26]. The INS velocity error is 60 m/min, the GSP deviation angle relative to the moving trihedron is $2\times 10^{-4}$ rad, the drift rate is $10^{-5}$ rad/min, and the sampling period is set to 1 min. The resulting adaptability degree of the GSP deviation angle relative to the moving trihedron is 100, and the adaptability degree of the GSP drift rate is 10,000.

When doubling the sampling period, the adaptability degree of the GSP deviation angle relative to the moving trihedron becomes 25, while the adaptability degree of the GSP drift rate decreases to 2500.

As the sampling period, T, increases, the degree of adaptivity decreases in accordance with Equations (23) and (24). This is consistent with physical considerations: the less frequently new information is received, the lower the adaptability degree.

When the aircraft’s flight altitude changes, the adaptability degree of the GSP deviation angle relative to the moving trihedron and the GSP drift rate vary with *g*.

Therefore, when the aircraft descends in the atmospheric flight phase, the adaptability degrees of these errors decrease.

The second method for calculating adaptability degree values (18) has a clearer physical meaning. The relative error in estimating the observed component of the state vector relative to the estimated nominal value in the case of estimating the deviation angle will be the same as the relative error in estimating the directly measured component after 100 min, and in the case of the drift rate, after 10,000 min.

The proposed adaptability degree criteria provide a quantitative assessment of adaptability for each state vector component of the system. In practical applications, this enables the development of models with desired adaptability properties.

In the case of a time-varying system matrix $\Phi_{k,k-1}$, the matrix *S* takes the following form:

(25) $$S_{k}=\left[\begin{array}{c} H\\ H\Phi_{k,k-1}\\ H\Phi_{k,k+1}\Phi_{k,k-1}\\ \cdots \end{array}\right]$$

Criteria (15), (16), and (18) remain unchanged, though the computation process for the referred noise coefficients becomes slightly more complex.

Additional verification was conducted using a semi-naturalistic experiment.

To test the performance of the presented scalar adaptive estimation algorithm with improved adaptivity characteristics, a semi-naturalistic simulation was conducted using a commercial INS installed on the test bench.

Let us consider how the accuracy of the adaptive scalar algorithm’s estimation of the INS speed determination error changes with increasing adaptivity of the algorithm’s model coefficients. The maneuvers of the base on which the INS is installed were simulated. The data from the semi-naturalistic experiment are presented in Table 1.

The results of INS modeling are presented. The error values obtained by the scalar adaptive estimation algorithm with model coefficient $a_{1}$ after 5 and 10 min of computation are presented. The INS error values obtained using the model coefficients $a_{2}$, which is characterized by an increased adaptivity characteristic determined by criterion (13); $a_{3}$, determined by criterion (15); and $a_{4}$, determined by criterion (16), are also presented. Estimation errors are presented as a percentage of the estimated value. The low accuracy of the INS error estimation is explained by the use of a scalar algorithm—only one component of the model state vector is taken into account. The advantage of scalar algorithms is their ease of implementation, which is very important when correcting INS for small-sized dynamic objects.

The results of a semi-naturalistic experiment demonstrated the viability of the developed algorithm. When changing the nature of the INS velocity estimation error, the accuracy of the 5 min estimation increased by an average of 5% compared to the prototype—a scalar adaptive algorithm without an adaptivity criterion. After 10 min, the accuracy became approximately the same and was maintained until the next maneuver of the base on which the INS is installed was simulated.

We will examine the INS errors in determining position obtained during the laboratory experiment. These errors should be compared with the calculated errors in determining latitude, which were calculated using the estimation algorithm (EA).The errors of a real INS mounted on a fixed base are primarily due to GSP drift. Therefore, the system errors caused by GSP drift are calculated. To calculate the INS errors in determining the distance traveled, we use the well-known formula:

(26) $$\delta x=-\varepsilon^{*}R\left(t-\frac{\sin\vartheta t}{\vartheta }\right).$$

Here, $\varepsilon^{*}$ is the constant GSP drift; ϑ=g/R. The drift estimates obtained by the EA must be averaged. The nature of the change in the horizontal GSP drifts over the laboratory experiment interval allows averaging over three intervals: 0–100, 100–200, and 200–325 min.

Thus, the INS error in determining the distance traveled will be

(27) $$\delta x=\varepsilon_{1}^{*}R\left(t-\frac{\sin\vartheta t}{\vartheta }\right)+\left(\varepsilon_{2}^{*}-\varepsilon_{1}^{*}\right)\left[\left(t-t_{1}\right)-\frac{\sin\vartheta (t-t_{1})}{\vartheta }\right]+\left(\varepsilon_{3}^{*}-\varepsilon_{2}^{*}\right)\left[\left(t-t_{2}\right)-\frac{\sin\vartheta (t-t_{2})}{\vartheta }\right].$$

To determine latitude and longitude, we use the formulas:

(28) $$\delta \varphi_{k}=\frac{\delta x}{R}$$

where δφ is the error in determining the latitude of a location; δx is the estimated eastern component of the error in determining the path of the real INS.

**Figure 2:**
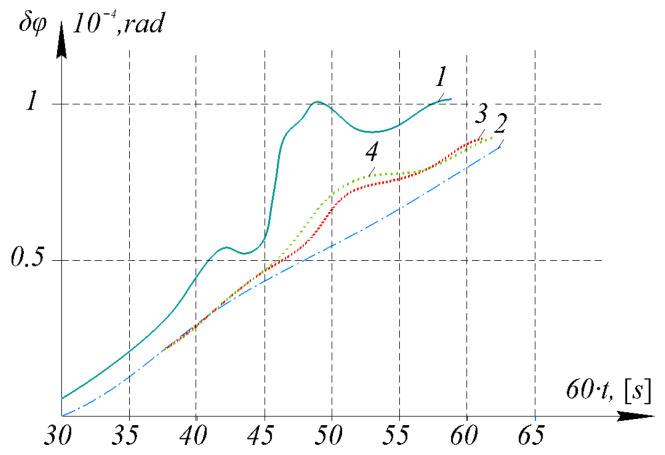
Errors in determining latitude in a real inertial navigation system and their estimates: 1—Errors in determining latitude obtained during a laboratory experiment; 2—Error estimates for the real system obtained using the adaptive navigation system with the coefficient $a_{1}$ of the classical model; 3—Error estimates for the real system obtained using the adaptive navigation system with the coefficient $a_{2}$; 4—Error estimates for the real system obtained using the adaptive navigation system with the coefficient $a_{4}$.

The calculated values of the latitude errors are comparable to the errors of the real INS in determining the position, which were obtained in a laboratory experiment. Modeling based on the data from the laboratory experiment with the real INS was performed using an EO with different values of the model coefficients of the process being evaluated. The results of the INS modeling are presented in Figure 2.

Errors in determining latitude become clearly apparent after 30 min of the experiment. A carrier maneuver is simulated over an interval of 40–50 min.

The results of the modeled error estimates for determining latitude showed a slightly worse result using adaptivity criteria compared to the EA estimates for determining velocity. This is due to the use of a special methodology that involves averaging GSP drift estimates and recalculating them using Formulas (26) and (27). Simulations showed that the best results in estimating the errors of a real INS in determining latitude were obtained using an EA with a coefficient $a_{4}$, obtained using criterion (18). Simulation results based on laboratory experiment data demonstrated the performance and higher accuracy of an EA with coefficients exhibiting a higher degree of adaptability during the maneuver execution phase.

## 5. Discussion of Results

The concepts of adaptability and observability degrees are proven to be closely related when implementing the proposed assessment approach. Their definitions are based on analyzing estimation accuracy and the time required to achieve specified accuracy. Therefore, the adaptability degree of state variables depends not only on model properties (determined by interconnections between state variables) but also on the efficiency of the estimation algorithm. The property of the estimation algorithm is characterized by the accuracy of the estimation.

The concepts of adaptability degree and state variable controllability degree [2,5] also demonstrate close similarity. A rapid response to a change in state while maintaining control accuracy is inherent in both well-controlled components of the state vector and is consistent with the idea of a high adaptability degree.

The distinctive feature of adaptability degree compared to the aforementioned qualitative characteristics of state variables is its prioritization of rapid response to changes and reduced emphasis on accuracy during response execution (whether in estimation or control). However, it is impractical to ignore the level of accuracy since the meaning of the process being implemented, the adaptability degree of which is being estimated, may be lost. The proposed criteria do not account for this reduced significance of estimation accuracy relative to the speed of achieving the target value. Therefore, employing the second criterion (18) seems appropriate—it evaluates the adaptability degree based on the number of computational steps required to reach a specified accuracy. Thus, the physical meaning is expressed more clearly. Although this criterion does not explicitly prioritize response speed as the dominant factor influencing adaptability degree, it allows for the specified estimation error variance to be increased. This approach enables partial assessment of a state variable’s response speed within the algorithm while preserving the operational validity of the process being implemented.

## 6. Conclusions

Two criteria for determining the adaptability degree of state variables in linear dynamic system models are proposed. It is assumed that these models are used in the estimation algorithm. Numerical criteria for quantifying state variable adaptability have been developed, and their application is demonstrated by assessing the adaptability degree of state variables in an aircraft INS error model.

The proposed criteria for calculating the adaptability degree of state variables in models are used to evaluate the qualitative characteristics of the models used in the estimation algorithms.

For further development of this research, future studies should establish approaches and specific criteria to assess adaptability degrees of nonlinear model state variables, which will enable their broader practical implementation.

## Figures and Tables

**Table 1:** Estimation errors with model coefficients calculated using different criteria.

*t* [min]	$a_{1}$	$a_{2}$	$a_{3}$	$a_{4}$
5	25%	21%	19%	21%
10	15%	15%	14%	15%

## Data Availability

The original contributions presented in this study are included in the article. Further inquiries can be directed to the corresponding author.
